# Tertiary structure prediction and identification of druggable pocket in the cancer biomarker – Osteopontin-c

**DOI:** 10.1186/2251-6581-13-13

**Published:** 2014-01-08

**Authors:** Subramaniam Sivakumar, Sivasitambaram Niranjali Devaraj

**Affiliations:** 1 Department of Biochemistry, Sri Sankara Arts and Science College, Enathur 631561, Tamilnadu, India; 2 Department of Biochemistry, University of Madras, Guindy Campus, Chennai 600025, Tamilnadu, India

## Abstract

**Background:**

Osteopontin (Eta, secreted sialoprotein 1, opn) is secreted from different cell types including cancer cells. Three splice variant forms namely osteopontin-a, osteopontin-b and osteopontin-c have been identified. The main astonishing feature is that osteopontin-c is found to be elevated in almost all types of cancer cells. This was the vital point to consider it for sequence analysis and structure predictions which provide ample chances for prognostic, therapeutic and preventive cancer research.

**Methods:**

Osteopontin-c gene sequence was determined from Breast Cancer sample and was translated to protein sequence. It was then analyzed using various software and web tools for binding pockets, docking and druggability analysis. Due to the lack of homological templates, tertiary structure was predicted using ab-initio method server – I-TASSER and was evaluated after refinement using web tools. Refined structure was compared with known bone sialoprotein electron microscopic structure and docked with CD44 for binding analysis and binding pockets were identified for drug designing.

**Results:**

Signal sequence of about sixteen amino acid residues was identified using signal sequence prediction servers. Due to the absence of known structures of similar proteins, three dimensional structure of osteopontin-c was predicted using I-TASSER server. The predicted structure was refined with the help of SUMMA server and was validated using SAVES server. Molecular dynamic analysis was carried out using GROMACS software. The final model was built and was used for docking with CD44. Druggable pockets were identified using pocket energies.

**Conclusions:**

The tertiary structure of osteopontin-c was predicted successfully using the ab-initio method and the predictions showed that osteopontin-c is of fibrous nature comparable to firbronectin. Docking studies showed the significant similarities of QSAET motif in the interaction of CD44 and osteopontins between the normal and splice variant forms of osteopontins and binding pockets analyses revealed several pockets which paved the way to the identification of a druggable pocket.

## Background

Cancer results from alterations that disrupt the appropriate controls and balances that direct normal cellular growth and development. These changes resulting in altered gene products or altered gene expression can occur in two classes of genes that interact with each other: genes that inhibit tumor suppressor genes and genes that facilitate cell growth and development [1]. Malignant tumors are characterized by dysregulated growth control, the overcoming of replicative senescence and the formation of metastases. Several growth factors and cytokines play pivotal roles in the regulation of proliferation, survival, adhesion and migration of neoplastic cells [2]. Decades of scrutiny into the molecular basis of cancer have largely focused on what causes oncogenic transformation and the incipient emergence of tumors [3]. The invasion of tumor cells is a complex, multistage process. To facilitate the cell motility, invading cells need to change the cell-cell adhesion properties, rearrange the extracellular matrix environment, suppress anoikis and recognize their cytoskeletons [4].

A biomarker is any substance, which when detected in biological samples or tissue, is associated with an increased risk of a disease. Serum biomarkers are produced by body organs or tumors and when detected in high amounts in the blood, can be suggestive of tumor activity. These markers are nonspecific for cancer and can be produced by normal organs as well. Most biomarkers are used infrequently for screening purposes. They are more often used to evaluate treatment effects or to assess the potential for metastatic disease in patients with established disease. Osteopontin (OPN) was identified as one such biomarker [5]. Osteopontin is a secreted glycoprotein that plays important roles in a wide range of biological processes, including tissue remodeling, inflammation, angiogenesis, tumor development and immunity to infectious disease [6]. Osteopontin also increases expression of HIF-1α through phosphatidyl inositol 3′–kinase/Acutely transforming retrovirus AKT8 in rodent T cell lymphoma (PI3-K/Akt) pathway [7].

The OPN is a 32.5-kDa multifunctional protein with multiple phosphorylation and glycosylation sites and contains an arginine-glycine-aspartic acid-binding (RGD) domain as well as two heparin-binding sites, one thrombin cleavage site (RSK [arginine-serine-lysine]) and a calcium-binding site. The protein functions as both a cell attachment protein and a cytokine that has a signaling function through the action of two cell adhesion molecules: αvβ3-integrin and CD44 [8]. It is also a tumor-associated protein, which mediates tumor transformation and malignant progression. OPN has been proposed to promote tumor progression through several mechanisms, including increased cell survival, migration, invasion, neovascularization, and modulation of immune function. The RGD domain of OPN functionally mediates cell adhesion, migration and invasion through integrin engagement. Interaction between the RGD domain of OPN and integrin receptors leads to Nuclear Factor-KappaB (NF-kB) and Focal adhesion kinase (FAK) actvation mainly through decreased apoptosis. These data indicate that the predominant mechanism, by which OPN promotes tumor growth and metastasis through the RGD domain, is enhancement of survival in the tumor microenvironment [9]. When OPN is cleaved at the RSK site by thrombin, it is separated into two approximately equivalent sized pieces, including N-terminal and C-terminal fragments. Thrombin is activated by tissue factor (TF) which is overexpressed on the surface of cancer cells. Both N-terminal and C-terminal fragments increases adhesion and migration of cancer cells through interaction with integrins and cyclophilin C respectively [10]. Enhanced OPN expression has been detected at the tumor site as well as in plasma and serum of patients with various types of cancers [11].

The existence in humans, of two osteopontin splice variants with deletions of exon 4 referred to as osteopontin-c or exon 5 called osteopontin-b and the normal osteopontin referred as osteopontin-a has been described by Young *et al*. [12]. Alternative splicing occurs in a region in a molecule that is upstream of the central integrin binding domain and the C-terminal CD44 binding domain. Interestingly, osteopontin-b expressed by transfection is unstable and the protein is degraded in the proteosome. In addition, osteopontin-b RNA is present at consistently low levels of expression in breast tissue specimens [13]. Osteopontin-a was found to be expressed in both normal and cancer cells to a lesser extent whereas osteopontin-c transcripts were never detected in the normal tissue samples but were present only in tumor cells [14]. The splice variant osteopontin-c, which does not contain the sequence encoded in exon-4, lacks an important domain for calcium induced aggregation and transglutamination. Lack of this domain forms the soluble form of the protein [15]. Among the three splice variants of osteopontin expressed in breast cancer, the shortest form, osteopontin-c, supports anchorage-independence more effectively than the full length form, osteopontin-a. Splice variant form, osteopontin-c, is brought about through the gain of function by the cancer cells, reflected in the activation of unique signal transduction pathways. Osteopontin-c coordinately induces oxidoreductase genes that are associated with the mitochondrial energy metabolism and with the hexose mono phosphate shunt [14].

Taken together, this growing list of studies suggests that osteopontin blood levels have a potential as a prognostic or diagnostic marker in prostate, breast, head and neck and other cancers. It should be noted, however, that osteopontin is unlikely to be a blood marker that is specific to cancer because osteopontin levels are also elevated in other conditions including sepsis, kidney disease and cardiovascular disease. But, the identification of the splice variant form of osteopontin-c solved this problem [14]. In order to study further about the role and function of osteopontin-c, the three dimensional structure might be useful, which is yet to be determined through x-ray crystallographic or NMR techniques. In this context, ***in silico*** structure prediction of osteopontin-c was carried out along with sequence analysis and docking studies.

## Methods

### Web based tools

The web based tools were used for the purpose of translation, similarity studies, tertiary structure prediction, model refinement, model evaluation, binding pockets prediction and docking. The list of websites along with web site addresses is shown in Table 1.

**Table 1:** Web based tool list

**S. No**	**Server**	**Website address**
1.	ExPASy Translate tool	http://web.expasy.org/translate/
2.	SignalP	http://www.cbs.dtu.dk/services/SignalP/
3.	PrediSi	http://www.predisi.de/index.html
4.	ClustalW	http://www.genome.jp/tools/clustalw/
5.	I-TASSER	http://zhanglab.ccmb.med.umich.edu/I-TASSER/
6.	SAVES Server	http://nihserver.mbi.ucla.edu/SAVES/
7.	SUMMA	http://silvio.cs.uno.edu/proteinrefinementserver/
8.	PocketFinder	http://www.modelling.leeds.ac.uk/pocketfinder/
9.	Q-Site Finder	http://www.modelling.leeds.ac.uk/qsitefinder/
10.	ClusPro	http://nrc.bu.edu/cluster/
11.	DoGSiteScorer	http://dogsite.zbh.uni-hamburg.de/

### Sequence source

Osteopontin-c gene sequence was determined from breast cancer sample and it was translated to protein sequence using ExPASy Translate tool (http://web.expasy.org/translate/) [16].

### Sequence analysis

Signal sequence of osteopontin-c was predicted using signalP [17] and PrediSi servers [18,19]. Tertiary structure prediction was carried out using I-TASSER tool [20]. Critical Assessment of Techniques for Protein Structure Prediction (CASP) is a community-wide experiment for testing the state-of-the-art of protein structure predictions which takes place every two years since 1994. The I-TASSER server (as “Zhang-Server”) participated in the Server Section of 7th (2006), 8th (2008), and 9th CASPs (2010), and was ranked as the No 1 server in CASP7, CASP8 and CASP9. Thus, this server selected for tertiary structure prediction. The c-score is a confidence score for estimating the quality of predicted models by I-TASSER. It is calculated based on the significance of threading template alignments and the convergence parameters of the structure assembly simulations. The c-score is typically in the range of (-5, 2), where a c-score of higher value signifies a model with a high confidence and vice-versa [20]. The quality of the predicted structure was examined using an online metaserver SAVES, which uses Procheck [21], WhatCheck [22], Verify3D [23], ERRAT [24] and PROVE [25] servers. The predicted structure was refined using SUMMA server [26]. Molecular dynamic analysis was carried out using GROMACS (GROningen MAchine for Chemical Simulations) software [27,28]. Structure visualization was carried out using Accelrys’ Discovery Studio Visualizer 1.7. Tertiary structure of osteopontin-a also predicted by I-TASSER server and subjected to other treatments as mentioned for osteopontin-c.

### Determination of conserved regions (domains)

In order to determine conserved regions (domains) in osteopontin-c of human, it was aligned with rabbit, cattle, chicken, house mouse, Norway rat and water buffalo osteopontin sequences using clustalW [29] at http://www.genome.jp/tools/clustalw/. RSK and RGD domain comparison was achieved by using Discovery Studio Visualizer (Accelrys Discovery Studio Visualizer, version 1.7, 2007; Accelrys Software Inc., San Diego). Tertiary structures of thrombin cleaved fragments were also predicted by I-TASSER server. The C-terminal fragment of osteopontin-c was used for hypothetical polymer formation using ICM Molsoft tool. Six subunits were utilized for the formation of polymer formation using import option of the ICM Molsoft tool.

### Docking

The predicted tertiary structures of osteopontin-a and osteopontin-c were docked with CD44 using Cluspro. ClusPro is the first fully integrated server that includes both docking and discrimination steps for predicting the structure of protein–protein complexes. The server can be used to discriminate a set of potential complex structures from several docking algorithms, or it can generate its own structures using DOT or ZDOCK [30].

### Binding pockets predictions

PocketFinder and Q-Site finder were utilized for binding pocket predictions [31]. PocketFinder is based on the Ligsite algorithm written by Hendlich *et al.*[32] which was used to predict small molecule binding sites in proteins. Q-Site finder uses the interaction energy between the protein and a simple van der Waals probe to locate energetically favourable binding sites.

### Druggable pocket predictions

DoGSiteScorer is an automated pocket detection and analysis tool which can be used for protein druggability assessment. Based on the three dimensional coordinates of a protein, its potential active sites on the protein surface are calculated with DoGSiteScorer. DoGSiteScorer is a grid-based function prediction method which uses a difference of Gaussian filter to detect potential pockets on the protein surface and splits them into subpockets. Subsequently, global properties, describing the size, shape and chemical features of the predicted pockets are calculated. Per default, a simple score is provided for each pocket, based on a linear combination on the three descriptors describing volume, hydrophobicity and enclosure. For the discrimination of the druggability, a subset of meaningful descriptors is used in a support vector maschine (libsvm). The druggability model was trained and tested on the druggable cavity directory dataset consisting of 1069 structures and yielded prediction accuracies of 88%. For each queried input structure, a druggability score between zero and one is returned. The higher the score the more druggable the pocket is estimated to be [33-35].

## Results and discussion

### Sequence analysis

Osteopontin-c gene sequence was determined from breast cancer sample and deposited to Genbank with ID JF412667. With the ExPASy Translate tool, a peptide sequence was deduced, consisting of 287 amino acid residues. This sequence was 100% identical to the protein sequence in GenPept database (NP_001035149.1). Both the signal prediction tools namely SignalP and PrediSi indicated the presence of a potential signal peptide in osteopontin-c protein. Signal sequence prediction servers predicted an N-terminal cleavage site between 16th and 17th amino acid residues of osteopontin-c sequence. After predicting the signal sequence, first 16 amino acid residues were identified as signal peptide and were removed from osteopontin-c sequence. The remaining protein sequence was utilized for tertiary structure prediction because during protein folding under in-vivo condition, the signal sequence is removed.

**Figure 1:**
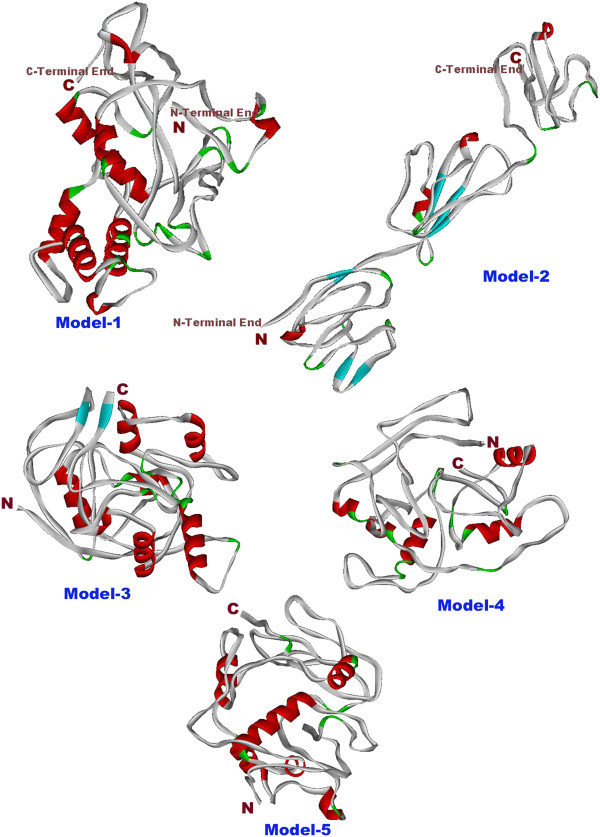
**Predicted structure of osteopontin-c by I-TASSER.** Figure shows five predicted models from I-TASSER Server. Of these five models, model 2 is reported as best model by comparing with the electron microscopic structure of Bone sialoprotein.

X-ray crystallography, NMR and cryo-electron microscopic studies were used in wet-lab for three dimensional structure predictions of proteins [36]. NMR studies carried out to predict three dimensional structure of only RGD tripeptide sequence of osteopontin [37]. Prediction of protein structure from amino-acid sequences has been one of the most challenging problems in computational structural biology for many years. Historically, protein structure prediction was classified into three categories: (i) comparative modeling, (ii) threading, and (iii) ab-initio folding. The first two approaches build protein models by aligning query sequences onto solved template structures. When close templates are identified, high-resolution models could be built by the template-based methods. If templates are absent from the Protein Data Bank (PDB) library, the models need to be built from scratch, i.e. ab-initio folding. This is the most difficult category of protein structure prediction. Such difficult task was attained using I-TASSER tool [20]. Tertiary structure of osteopontin-c was predicted using I-TASSER Server [38]. Predicted tertiary structure is shown in Figure 1. I-TASSER result provided five models with different c-scores, cluster density and number of decoys as shown in Table 2.

**Table 2:** I-TASSER scores for predicted models of osteopontin-c

**S. No**	**Model**	**C-Scores**	**No. of decoys**	**Cluster density**
1.	Model 1	-3.99	703	0.0237
2.	Model 2	-3.56	544	0.0364
3.	Model 3	-3.99	209	0.0237
4.	Model 4	-4.61	199	0.0127
5.	Model 5	-4.43	179	0.0151

Predicted model-2 contains the highest c-score and also showed high similarity with electron microscopic structure of bone sialoprotein (BSP), which belongs to same Small integrin-binding ligand N-linked glycoproteins (SIBLINGs) protein family. Both the Predicted model-2 and bone sialoprotein were found to have thread with globular domain structure. Electron crystallography is a form of microscopy that uses a beam of electrons to construct images of small solids such as proteins. This process is used to determine and predict the structure and arrangement of a protein from secondary structure crystals such as alpha helices or beta sheets based on electron scattering. By electron crystallography method BSP structure determined. The BSP is a monomer possessing a globular structure with a diameter of 10 ± 1 nm that is linked to a thread-like structure of 25 ± 6 nm length. The globule is likely to correspond to the C-terminal part and the threadlike structure to N-terminal part of the protein [39].

Small integrin-binding ligand N-linked glycoproteins (SIBLINGs), a family of five integrin binding glycophosphoproteins comprising osteopontin (OPN), bone sialoprotein (BSP), dentin matrix protein 1 (DMP1), dentin sialophosphoprotein (DSPP) and matrix extracellular phosphoglycoprotein (MEPE), are an emerging group of molecular tools that cancer cells use to facilitate their expansion. SIBLINGs are soluble, secreted proteins that can act as modulators of cell adhesion as well as autocrine and paracrine factors by their interaction with cell surface receptors such as integrins. BSP and OPN are two members of the SIBLING family of genetically related proteins that are clustered on human chromosome 4. These two proteins have several common binding partners like CD44, integrins, matrix metalloproteinases (MMP), and complement factor H (CFH). Because of that, they had common interaction domains like RGD and in turn structure [40,41].

Predicted tertiary structure of osteopontin-c had three domains, namely N-terminal domain, central domain and C-terminal domain. RGD and RSK motifs and two helical regions and three turns were present in central domain. N-terminal end domain consists of four antiparallel sheets, two helical regions and five turns. C-terminal end domain consists of one sheet, one helical region and one turn. Earlier hypothetical structure for osteopontin was predicted by Ganss. It was an open extended and flexible structure. Model-2 of I-TASSER result supported the proposal of Ganss [42].

Model-2 was refined using SUMMA Server. The predicted structure was refined by fixing side chains, fixing problematic loops, removal of amino acid clashes (bumps) and energy minimization. Potential functions used in structure prediction and refinements are typically grouped into two general classes: traditional “physical” molecular mechanics potentials and statistically derived 'knowledge-based” potentials [26]. The refinements did not yield any drastic change in the initial predicted structure augmenting the correctness of the predicted structure, which was confirmed by superimposition studies.

**Figure 2:**
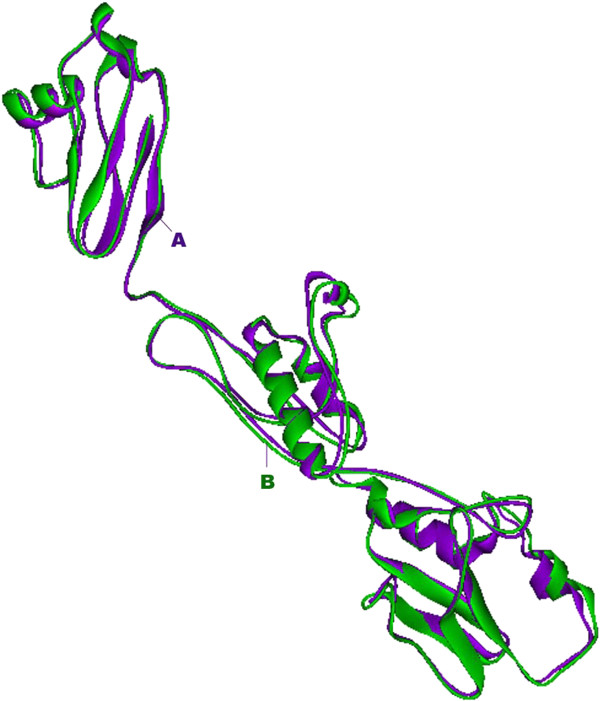
**Superposition comparison for predicted and refined structure of osteopontin-c.** Here all conformations are superimposed with a reference structure (Predicted model) using RMSD fit. Accelry’s Discovery Studio Visualizer 1.7 was utilized for the superimposition. **A**. predicted structure. **B**. Refined structure.

Refined structure and predicted structure were superimposed using Accelry’s Discovery Studio Visualizer 1.7 software. RMSD value for the superimposition was found to be 0.92 A°. The superimposed structure is shown in Figure 2. It was found that the structure of osteopontin-c was similar in structure to fibronectin which was determined by Amit sharma *et al*., [43]. Fibronectin (FN) is a large glycoprotein found on cell surfaces, in the connective tissue matrix and in extracellular fluids. It participates in cell adhesion, spreading, migration, extracellular matrix formation, hemostasis and thrombosis. FN binds to fibrin, collagens, gelatin, DNA, integrins, heparin and proteoglycans. Due to the common interacting partners both have similar structures [43].

**Figure 3:**
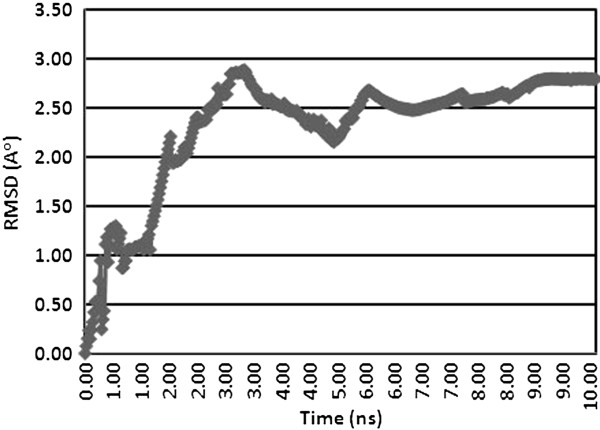
**The Root mean square deviation plot (Carbon Alpha back bone) obtained from GROMACS tool during molecular dynamics simulation for 10 nanoseconds.** Root mean square deviation (RMSD) of osteopontin-c model during the molecular dynamics (MD) simulation. The stabilization of the structure occurred in approximately 10 ns. Generated by GROMACS.

SAVES validation server results showed that refined structure was slightly improved from the predicted structures. Comparisons of validation server results are listed in the Table 3. Lesser values in ProCheck, WhatCheck and PROVE servers result of refined structure showed that the structure quality was improved. Verify 3D values were found to be insignificant. Higher value in ERRAT result also supported the structure refinement process. Molecular dynamics (MD) is a computer simulation of physical movements of atoms and molecules [44]. Molecular dynamic simulation with explicit waters for 10 nanoseconds was assessed by GROMACS software [45]. The root-mean-square deviation (RMSD) variations for the molecular dynamic simulation are given in the Figure 3. It clearly showed that osteopontin-c had average RMSD deviation of 2.79 A°. Anyway, the refined structure was found to be an improved one. But one cannot reject predicted structure of osteopontin-c because the structure does not showed drastic variation in tertiary structure during molecular dynamic simulations. RMSD values below 5–6 A° are generally considered being characteristic for a stable protein in molecular dynamics simulations [46].

**Table 3:** Significant SAVES validation server results of osteopontin-c

**S. No**	**Servers**	**Parameters**	**Before refinement**	**After refinement**
1.	PROCHECK*	G-Factor Value	- 0.23	- 0.33
2.	WhatCheck*	Bond Lengths	1.257	0.782
3.	Verify3D*	3D – 1D score > 0.2	39.93%	43.86%
4.	ERRAT^#^	Overall Qualaity	36.55%	74.812%
5.	PROVE*	% Outliers	12.30%	10.2%

“*” - Lesser value indicates better quality.

“#” - Higher value indicates better quality.

### Determination of conserved regions (domains)

**Figure 4:**
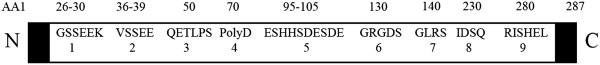
Selected structural domains and their corresponding locations in the human osteopontin-c.

**Figure 5:**
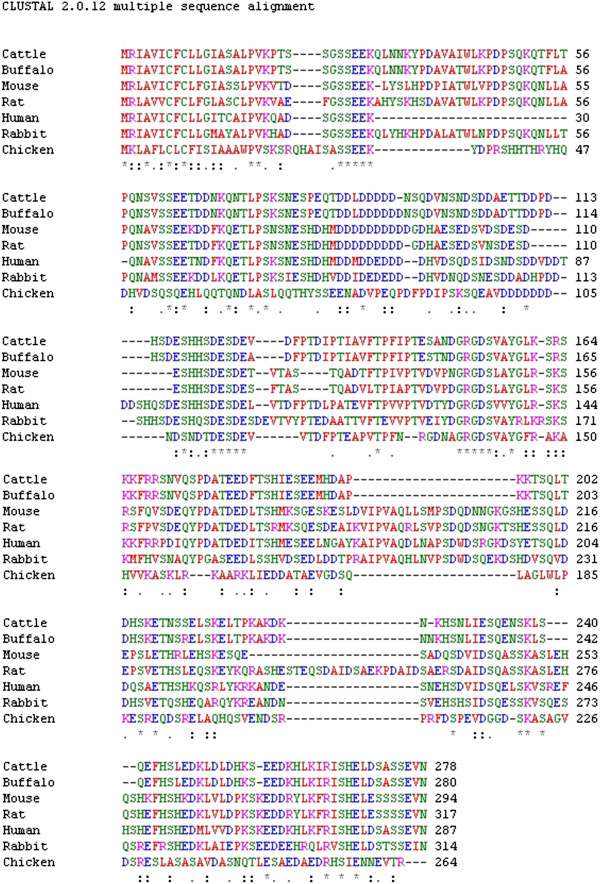
**Multiple sequence alignment of osteopontin.** Multiple sequence alignment of osteopontin amino acid residues of *Bos taurus* (cattle), *Bubalus bubalis* (water buffalo), *Homo sapiens* (human), *Oryctolagus cuniculus* (rabbit), *Mus musculus* (mouse), *Rattus norvegicus* (Norway rat) and *Gallus gallus* (chicken). The *(star) in the sequence represents identical residues.

**Figure 6:**
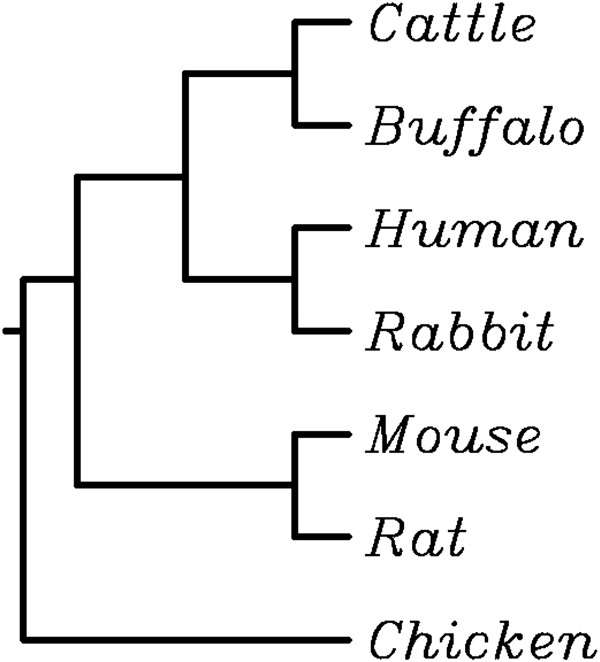
**Phylogenetic tree.** Phylogenetic tree obtained using a multiple alignment of osteopontin protein sequences from cattle (NP_776612), water buffalo (ABD73011), human (NP_001035149.1), rabbit (NP_001075663), mouse (NP_033289), rat (NP_037013) and chicken (NP_989866). See text for details.

The amino acid sequences of osteopontin were derived from human [47], mouse [48], rat [49], rabbit [50], Water buffalo [51] and cattle [52]. Multiple sequence alignment was carried out using ClustalW program at Eurobean Bioinformatic Institute. Based on sequence alignments, the amino acid sequence was divided into nine parts which were represented in Figure 4. Of these nine parts, only five parts have known functions i.e. PolyD – Binds with calcium, RGD – Integrin binding site, GLRS – Thrombin cleavage site, Eighth part – Calcium Binding site, and Ninth part- Heparin Binding site. Of these conserved regions, Poly D (4th), GRGDS (6th) and GLRS (7th) regions are well known for their functions [53]. Human and Rabbit sequences were found to have 64% similarity whereas human and chicken were found to have only 21% similarity score in multiple sequence alignment. Multiple sequence alignment is shown in Figure 5. Phylogenetic relationships between these organisms are shown in Figure 6. Distinct differences were found to be present between human and chicken which could reflect functional and developmental differences between chicken and mammalian osteopontins [54].

**Figure 7:**
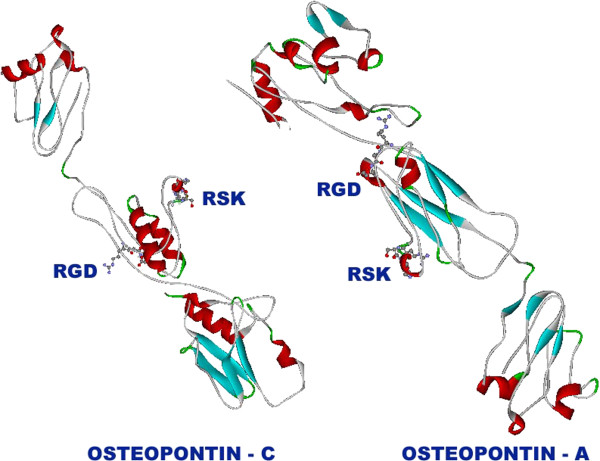
**RGD and RSK domains of osteopontin-c and osteopontin-a.** RGD and RSK domains are represented in ball and stick model whereas remaining part of the protein is represented in solid ribbon model using Accelry’s Discovery Studio Visualizer 1.7.

**Figure 8:**
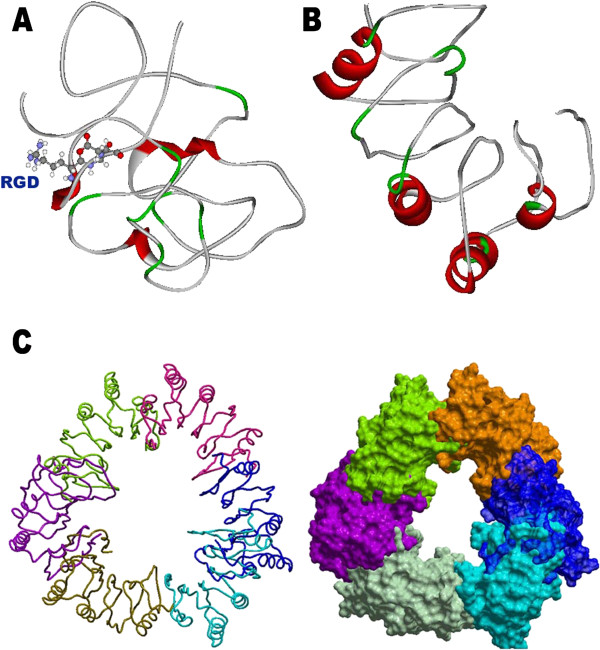
**N-terminal fragment (A) and C-terminal fragment (B) of osteopontin-c after the action of thrombin.** Polyimerized structure **(C)** of C-terminal fragment of osteopontin-c. RGD domains are represented in ball and stick model in N-terminal fragment. Alternate presence of helices might be seen in C-terminal fragment which might provide the clue for the formation of pore or fibre. Tube and surface model are represented in the Figure C. Polymerization was achieved using ICM Molsoft Tool.

Predicted osteopontin-a and osteopontin-c contain both RGD and RSK motifs which can be seen in Figure 7. Difference in the role of osteopontin-c from osteopontin-a in cancer can be clearly seen from Figure 7 that is the exposure of RSK domain. Due to the exposure of RSK domain, thrombin can easily cleave osteopontin-c into two fragments N-terminal and C-terminal fragments. The fragments were then subjected to tertiary structure prediction. Predicted structures of N-terminal and C-terminal fragments are shown in Figure 8. Predicted structure showed totally different structure when compared with the intact osteopontin-a and osteopontin-c. This might be the essential reason for the role of osteopontin-c in cancer progression and metastasis. C-terminal fragments’ polymer might form channel like structures which is shown in Figure 8. Presence of RGD and RSK motifs in osteopontin were reported by several studies [55,56]. Thrombin cleaves between R and S residues of RSK sequence [55,56]. This cleavage occurs within six amino acid residues of the GRGDS sequence, raising the interesting possibility that thrombin-cleavage further activates osteopontin by allowing greater accessibility of the GRGDS domain to cell surface receptors [57-60]. Xuan *et al.*[61] have shown that thrombin cleavage of osteopontin abolishes its cell binding function. The N-terminal GRGDS-containing osteopontin thrombin-cleavage fragment is highly active in promoting tumor cell migration [62]. The N-terminal fragment contains two integrin binding sites. These integrin binding domains include a SVVYGLR domain and a well-characterized RGD domain [63,64]. Furthermore, a recent study by Mi *et al*. [65] demonstrated that the thrombin-cleaved COOH-terminal fragment of osteopontin can activate downstream signaling and influence breast cancer cell migration and invasion in-vitro. The COOH-terminal fragment of osteopontin binds with another marker of metastatic function (cyclophilin C or rotamase) to the CD147 cell surface glycoprotein (also known as extracellular matrix metalloproteinase inducer or EMMPRIN), to activate Akt1/2 and matrix metalloproteinase-2 [65,66].

**Figure 9:**
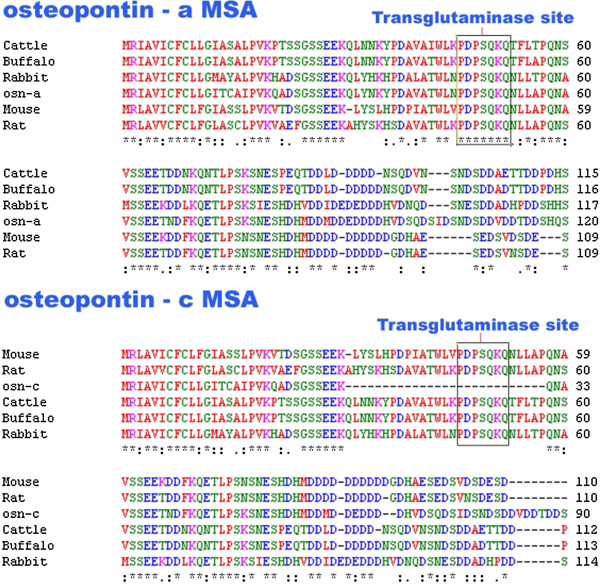
**Multiple sequence alignment comparison between osteopontin-a and osteopontin-c with other species with respect to transglutaminase acting stie.** Alignment analysis clearly showed the absence of transglutaminase site in osteopontin-c. Transglutaminase-2 acting site is indicated by an open box.

Previous studies in bovine osteopontin clearly demonstrated that gln-x-gln sequence is required for transglutaminase activity of cross linking osteopontin through gamma-glutamyl-epsilon-lysino peptidyl bond formation [67]. Multiple sequence alignment in Figure 9 clearly showed that the transglutaminase acting site is not present in osteopontin-c. The probable site is present between 46th and 52nd residues (PDPSQKQ) in osteopontin-a. The absence of this region in osteopontin-c clearly showed that transglutaminase will not act on it. This might be the essential reason for the pathological role of osteopontin-c and it may not be crosslinked with extracellular matrix and thus it results in cell migration. This fact was also supported by the lack of exon-4 expression in osteopontin-c by Weber [15].

### Docking

**Figure 10:**
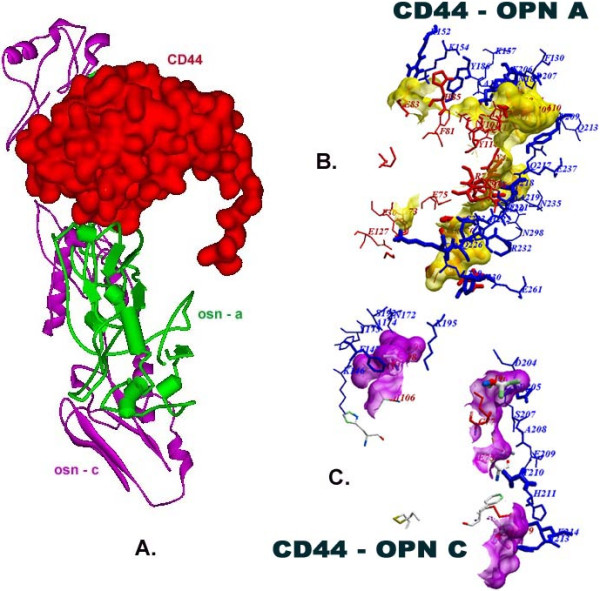
**Docked structure of CD44 with osteopontin-c and osteopontin-a. A**. Docked structure of CD44 (red) and osteopontin-a (green) and osteopontin-c (purple). **B**. Interaction site of CD44 (red) and osteopontin-a (blue). **C**. Interacation site of CD44 (red) and osteopontin-c (blue). It was achieved using Accelry’s Discovery Studio Visualizer 1.7 Tool.

Interestingly, OPN binding to CD44 results in the propagation of cytosolic signals that enhance integrin activation and thus migration in cancer cells. Binding of OPN with CD44 actively promote local proteolysis through binding with MMP3 and also activate complement factor H (CF-H) which protects cancer cell from complement mediated lysis. Both integrins and CD44 have well-established roles in tumor progression. Therefore, interfering with these receptor–ligand interactions by controlling receptor cell surface expression, blocking receptor–ligand binding or suppressing associated signal transduction are promising ways to block both tumor development and metastatic dissemination [40]. Thus, CD44 is selected as a potential receptor for docking studies. Docking analysis was carried out using CD44 as receptor and osteopontin types osteopontin-a and osteopontin-c as ligands. Docking results were analyzed manually and interaction sites were analyzed using Discovery Studio Visualizer 1.7 neighbour analysis tool. The results of ClusPro docking studies between CD44 and osteopontin-a and osteopontin-c showed for the first time that both of them interact in a similar site. Interaction of osteopontin to CD44 was proved by many studies [39,68] but none of the previous studies determined the interaction sites. Docked structures of CD44 and osteopontins and the interaction domains of CD44 and osteopontins were shown in Figure 10. It was identified that aspargine (233rd residue), serine (234th residue) and threonine (237th residue) residues were essential for interactions in osteopontin isoform sequences. These residues might form the QSAET motif essential for the interaction of CD44 and osteopontins which is reported first time in the present study. Of these three residues, serine and threonine were found to be highly conserved in various species as was evident from the multiple sequence alingment. As the outcome, it was found out that serine and threonine residues are essential for interaction with CD44. This result supports the result of serine (234th residue) residue being glycosylated as a post translational modification in human osteopontin [69].

### Binding pockets prediction

**Figure 11:**
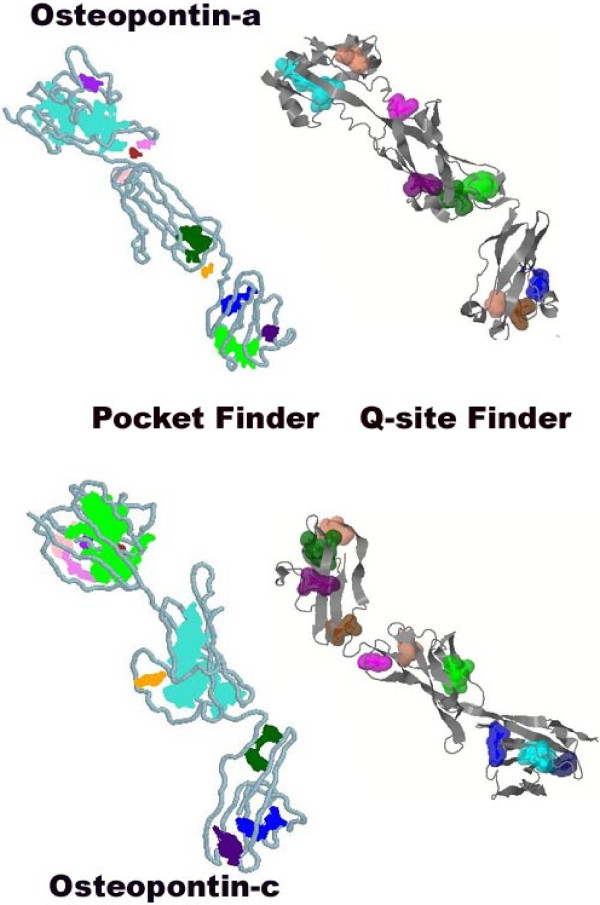
**Predicted binding pockets in osteopontin-c and osteopontin-a.** PocketFinder and Q-site Finder predicted pockets in osteopontin-a and osteopontin-c are shown as groups.

Binding pockets prediction is an essential step towards drug designing and docking studies [70]. Predicted binding pockets are shown in Figure 11. Pocket Finder detected ten pockets in both osteopontin-a and osteopontin-c. Eighth pocket of osteopontin-a was found to be present in the close region of RSK motif whereas no pocket contains RGD motif. First pocket of osteopontin-c was found to contain both RSK and RGD motifs. This result again confirms the role of osteopontin-c in cancer biology with respect to RSK and RGD motifs. Q-site finder predicted ten binding pockets from both osteopontin-a and osteopontin-c. Osteopontin-a has two pockets, namely, sixth and seventh, which contain RSK and RGD motifs in its outer layer, respecively. Osteopontin-c was found to have second and seventh pockets with RGD and RSK motifs, respectively. From all these results, the main novelty identified was that osteopontin-c contains binding pockets with highest rank with RGD and RSK motif, whereas osteopontin-a does not. Osteopontin promotes tumor growth and metastasis through the RGD domain [9].

### Druggable pocket identification

Pockets are ranked according to interaction energy, and it is assumed that these relate to locations where a putative ligand could bind and optimise its van der Waals interaction energy. The total energy of the pocket defines its ability to bind a small molecule and therefore its druggability [71]. Druggable pockets differ from binding pockets because druggable pocket was predicted using the druggable cavity directory dataset of drugs. A druggable pocket in osteopontin-c was predicted by DoGSiteScorer [34] and manual inspection of the pocket with their energies with the help of Swiss PDB Viewer 3.7 tool. It was found that the Q-site finder predicted eighth pocket with APSD (170th to 173rd residues) and TSQLD (184th to 188th residues) motifs can be used as a drug target in osteopontin-c due to the presence of pocket with maximum energy and druggable score. Amino acid residues of this predicted pocket was found to be conserved which was proved by multiple sequence analysis [Figure 5]. Elevated expression of osteopontin-c has been found in many cancers and the level of its expression is associated with the metastatic potential of cancer. Thus targeting osteopontin using the druggable pocket would be a logical approach for cancer management [72].

## Conclusion

The key finding of the present study is the discovery, for the first time, of the binding site of CD44 in osteopontin-c which has aspargine (233rd residue), serine (234th residue) and threonine (237th residue) residues. During the course of the study, a novel druggable pocket with APSD and TSQLD motifs was also found, which will be useful for future computer aided drug designing studies. Another important finding is that the RSK sequence is exposed to thrombin in osteopontin-c splice variant only as evidenced by the predicted tertiary structure, which explains the fact that only osteopontin-c is involved in metastasis. Due to the action of thrombin, osteopontin-c is fragmented into N-terminal and C-terminal fragments easily. Hypothetical proposal for the formation of channel like conformation by osteopontin-c was achieved with help of C-terminal fragment and favors cancer cell migration and metastasis. Absence of “PDPSQKQ” sequence in osteopontin-c avoids full length protein polymerization by transglutaminase-2 and favors metastasis. On the other hand, full length osteopontin-a is polymerized by transglutaminase-2 and thus, osteopontin-a cannot help metastasis because polymer favors cell adhesion. Obviously, experimental elucidation might be useful for further validation of real time tertiary structure of osteopontin-c. Until then, the present predicted structure might be used for computational drug design for osteopontin-c with respect to prevention of cancer.

## Competing interests

The authors declare that they have no competing interests.

## Authors’ contributions

Authors SS and SND, carried out sequence analysis and structure prediction studies, participated in docking and molecular dynamic analysis and drafted the manuscript. Both authors read and approved the final manuscript.
